# Enhancing motivation with the “virtual” supervisory role: a randomized trial

**DOI:** 10.1186/s12909-015-0348-8

**Published:** 2015-04-14

**Authors:** Majken T Wingo, Kris G Thomas, Warren G Thompson, David A Cook

**Affiliations:** 1 Department of Medicine, Division of Primary Care Internal Medicine, Mayo Clinic College of Medicine, 200 First Street SW, Rochester, MN 55905 USA; 2 Department of Medicine, Division of Preventive, Occupational, and Aerospace Medicine, Mayo Clinic College of Medicine, 200 First Street SW, Rochester, MN 55905 USA; 3 Department of Medicine, Division of General Internal Medicine, Mayo Clinic College of Medicine, 200 First Street SW, Rochester, MN 55905 USA

**Keywords:** Motivation enhancement, Task value, Instructional design, Test performance

## Abstract

**Background:**

We aimed to explore the influence of a motivationally-enhanced instructional design on motivation to learn and knowledge, hypothesizing that outcomes would be higher for the enhanced instructional format.

**Methods:**

Medicine residents completed four online learning modules on primary care topics. Using a crossover design, learners were randomized to receive two standard and two motivationally-enhanced learning modules. Both formats had self-assessment questions, but the enhanced format questions were framed to place learners in a supervisory/teaching role. Learners received a baseline motivation questionnaire, a short motivation survey before and after each module, and a knowledge posttest.

**Results:**

One hundred twenty seven residents were randomized. 123 residents (97%) completed at least one knowledge posttest and 119 (94%) completed all four posttests. Across all modules, a one-point increase in the pretest short motivation survey was associated with a 2.1-point increase in posttest knowledge. The change in motivation was significantly higher for the motivationally enhanced format (standard mean change −0.01, enhanced mean change +0.09, difference = 0.10, CI 0.001 to 0.19; p = 0.048). Mean posttest knowledge score was similar (standard mean 72.8, enhanced mean 73.0, difference = 0.2, CI −1.9 to 2.1; p = 0.90).

**Conclusions:**

The motivationally enhanced instructional format improved motivation more than the standard format, but impact on knowledge scores was small and not statistically significant. Learners with higher pre-intervention motivation scored better on post-intervention knowledge tests, suggesting that motivation may prove a viable target for future instructional enhancements.

## Background

A learner’s motivation can be a significant determinant in overall academic achievement. Medical educators strive to teach effectively, but unmotivated trainees may not acquire knowledge as well as trainees with higher drives to learn [1]. Since motivation is a learner characteristic, teachers often question what they can do to improve motivation from an instructional standpoint. Previous studies on motivation have targeted learner groups with limited relevance to medical education [2-6]. Incorporation of or access to online platforms has improved motivation in college students [2,3]. In one study of a computer-aided learning environment, video-based instruction had a more motivating effect than text-based instruction [4]. Granting a learner’s choice in training can improve motivation in adults [5], and in children, putting problems into fantasy contexts can improve motivation and learning [6]. Suggestions to enhance motivation among health professions trainees have been proposed [7], but the most effective mechanisms to increase motivation to learn in this population are unknown.

Many models of motivation for learning incorporate some variation of two core concepts, namely expectancy (the extent to which the learner expects to succeed) and value (the importance of learning to that learner). The expectancy-value theory of motivation suggests that the expectation of success positively influences the learner’s perception of learning importance. Expectancy is influenced by a learner’s perceptions of his or her own competence, goals, and the expected difficulty of the task [8]. Task value encompasses learners’ perception of task importance, interest in the task, and their perception of the task’s relevance to their future goals [9]. In non-medical education research, task value perceptions predicted college students’ midterm scores [10], and high school students were more likely to learn mathematics and less likely to avoid mathematics when they perceived mathematics as offering high task value [11]. Among medical students, low-performing students have reported lower task value beliefs [12], and task value beliefs have shown positive associations with academic achievement [13]. There has been a call for increasing research on motivation in medical education, but as Kusurkar described in 2012, medical education literature lacks publications that link curriculum development to stimulation of learner motivation [1,14]. In this study, we aimed to explore the influence of a motivationally-enhanced instructional design on motivation to learn and knowledge scores, hypothesizing that outcomes would be higher for the enhanced format.

Empiric evidence does little to inform efforts to directly enhance motivation to learn. However, it is commonly accepted that being required to teach a topic enhances both motivation and learning, and some theories of instruction bear this out [15,16]. Limited research corroborates the idea that putting learners into a teaching or supervisory role will enhance motivation and/or learning. For example, one study found that peer tutoring activity correlated with higher motivation among medical students [17]. Other studies have found that residents given formal teaching responsibilities showed improved learning of a given topic compared to lectures or self-study [18], and that residents perceive that teaching medical students improves their own learning [19,20]. Based on these promising findings, we hypothesized that placing residents in a “virtual” teaching role - that is, in the context of supervising a medical student - would enhance motivation and learning.

## Methods

To evaluate this hypothesis we conducted a randomized crossover trial comparing standard and motivationally-enhanced online learning modules in a course for medicine residents.

### Setting and sample

This study was undertaken at an academic medical center between November 2010 and June 2011. There were 168 residents (144 internal medicine, 24 family medicine) in the Mayo School of Graduate Medical Education in Rochester, Minnesota eligible to participate in the study. The study was deemed minimal risk and exempt after Mayo Clinic IRB review. Prior to study initiation, informed consent was obtained. As part of the consent process, participants were informed that the study would “compare two module formats to see if one format promotes more effective learning than the other”.

### Interventions

The Internal Medicine Ambulatory Care curriculum included four online learning modules that covered primary care topics: hypertension, obesity, coronary artery disease, and chronic obstructive pulmonary disease. These modules were updated to include current guidelines and evidence for each condition. Modules were released at approximately two-week intervals, and residents could finish available modules in any order. Each module contained text, images, hyperlinked resources, and case-based self-assessment questions. The learning modules were created using Articulate Presenter (www.articulate.com), a program that creates Flash presentations from PowerPoint slides. Both module formats consisted of didactic information with a total of 10 to 12 self-assessment questions interspersed throughout the module. The standard format self-assessment questions were typical case-based knowledge questions. The motivationally-enhanced format included the same question content, but the clinical cases were framed to have residents imagine themselves supervising a medical student in clinic. Response options were changed from a list of management approaches (standard format) to a list of responses and rationale one might use in a teaching role (enhanced format). Table 1 provides an example of each question format. Both formats provided the answer and explanation to the question immediately after the resident provided a response.

**Table 1:** **Self-assessment question formats**

Standard format	Enhanced format
A 72 year old man presents for evaluation of chest pain. He has a history of 3-vessel CABG about 8 years ago, and he has been doing very well up until now. Over the past three months he has noted typical angina near the end of his daily two-mile walk. He also has moderate COPD, for which he uses albuterol with good effect, and hypertension. Medications include metoprolol, aspirin, lovastatin, and lisinopril. Exam reveals femoral bruits and decreased pedal pulses.	**You are supervising a medical student in continuity clinic.** A 72 year old man presents for evaluation of chest pain. He has a history of 3-vessel CABG about 8 years ago, and he has been doing very well up until now. Over the past three months he has noted typical angina near the end of his daily two-mile walk. He also has moderate COPD, for which he uses albuterol with good effect, and hypertension. Medications include metoprolol, aspirin, lovastatin, and lisinopril. Exam reveals femoral bruits and decreased pedal pulses.
The most appropriate next step in management is:	**The medical student recommends an exercise ECG (treadmill exercise test). The most appropriate response is**:
a) Exercise ECG (treadmill exercise test)	**a) That’s a good plan**
b) Exercise sestamibi	**b) That’s not the best plan; we should order an exercise sestamibi because he has had CABG**
c) Adenosine sestamibi	**c) That’s not the best plan; we should order an adenosine sestamibi because he has femoral bruits and diminished pedal pulses**
d) Dobutamine echocardiogram	**d) That’s not the best plan; we should order a dobutamine echocardiogram because he has COPD and diminished pedal pulses**

Differences are in bold. Enhanced format placed the learner in a supervisory role. Single-best-answer multiple-choice questions were used in pairs matched for content. Feedback was identical for both formats.

Feedback: In patients such as this, who have had coronary revascularization (either surgical or percutaneous), imaging studies are preferred over non-imaging studies. This patient does not appear to have significant limitation of physical activity, so exercise testing would be preferred. Exercise sestamibi would probably be the best choice given COPD, although an exercise echocardiogram would also be a reasonable option.

### Randomization

**Figure 1:**
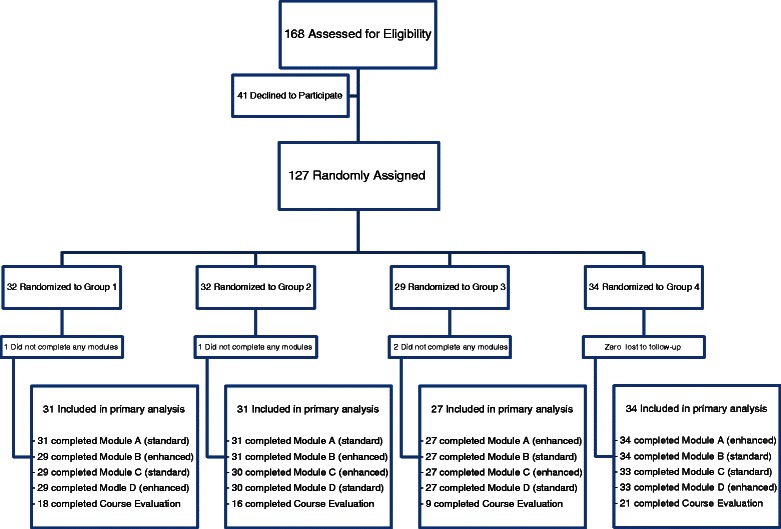
**Participant flow diagram.**

Residents who chose to participate completed two modules in the standard learning format and two in the motivationally-enhanced format. Participants were randomly assigned to one of four groups, with each group following a different sequence of module formats in a crossover exposure design (see Figure 1). MINIM (https://www-users.york.ac.uk/~mb55/guide/minim.htm) was used for randomization with stratification by post-graduate year (PGY) and continuity clinic site. Participant consent was obtained before group allocation. Residents who declined to participate in the study received all four modules in the standard format.

### Instruments and outcomes

Primary outcome measures were motivation (measured at baseline and then before and after each module) and posttest knowledge score. Secondary outcome measures included time, mental effort, and perceptions of which format was more efficient, more effective for learning, more motivating and overall preferred.

To measure baseline motivation, all residents completed the Motivated Strategies for Learning Questionnaire (MSLQ) prior to beginning their first learning module [21]. This 31-question instrument uses a 7-point scale (1 = not true of me, 7 = very true of me) and has validity evidence for use in this context [22]. Since motivation may vary by topic, residents also completed a short 13-question motivation inventory immediately before each learning module. The pre-module short motivation inventory was adapted from the MSLQ by selecting task value and self-efficacy questions that had high factor loadings from a previous factor analysis study [22]. The post-module short motivation inventory included task value questions (the same task value questions as in the pre-module motivation inventory), and single questions about time to complete each module, mental effort, and time elapsed between completing the module and starting the posttest. We collected information on gender, training program, and post-graduate year.

After completing each module, residents took a knowledge posttest. The test consisted of 14 to 18 (depending on the module) multiple choice questions adapted based on formal item analysis from questions used in previous research [23].

After finishing all four modules and posttests, residents completed a course evaluation survey containing questions about module format preference, efficiency, effectiveness, and which was more motivating (“which format did you prefer/find more efficient/find more effective/which format best motivated you to learn?”). These items used a 7-point scale (1 = strongly prefer standard format, 7 = strongly prefer enhanced format).

### Statistical analysis

Posttest knowledge scores, motivation scores, time spent, and mental effort were compared between formats using mixed linear models that accounted for repeated measures and for differences between modules. Additional adjustments included residency program, post-graduate year, and gender. We analyzed format preferences using the Wilcoxon signed rank test (testing whether results differed from the scale median). Of 168 residents eligible to participate, we estimated 75% participation and 20% drop-out leaving an anticipated 100 residents to complete the study. Using an expected standard deviation of 10 percentage points, 100 subjects would provide 85% power to detect a difference of 3 points (effect size 0.3) on knowledge tests, which we felt to be a minimum meaningful difference. All individuals were analyzed in the groups to which they were randomly assigned. All analyses used two-sided alpha error of 0.05. All analyses were done using de-identified data.

## Results

One hundred twenty seven residents gave consent to participate and were randomized. One hundred twenty three residents (97%) completed at least one knowledge posttest, 119 (94%) completed all four posttests, and 64 (50%) completed the course evaluation survey as summarized in Figure 1. Table 2 summarizes demographic data. Internal consistency for the knowledge posttest was good (Cronbach’s alpha 0.76). Statistical adjustments for training program, gender, post-graduate year, and time between module completion and posttest did not change any of the study findings reported below (results not shown).

**Table 2:** **Participant demographics**

Characteristic	Response	Group 1*	Group 2	Group 3	Group 4
		N = 32	N = 32	N = 29	N = 34
Gender, No. (%)	Female	14 (44)	17 (53)	10 (34)	17 (50)
Residency Program, No. (%)	Internal Medicine	28 (88)	28 (88)	25 (86)	29 (85
	Family Medicine	4 (12)	4 (12)	4 (14)	5 (15)
Post-Graduate Year, No. (%)	PGY1	11 (34)	14 (44)	10 (34)	11 (32)
	PGY2	12 (38)	9 (28)	11 (38)	11 (32)
	PGY3	9 (28)	9 (28)	8 (28)	10 (29)
MSLQ Score, mean (standard deviation)	Motivation Domain	Group 1	Group 2	Group 3	Group 4
	Intrinsic goal	5.4 (0.8)	5.4 (1.0)	5.7 (0.7)	5.2 (1.0)
	Extrinsic goal	4.4 (1.2)	4.1 (1.2)	4.4 (1.2)	4.0 (1.2)
	Task value	5.7 (0.7)	5.4 (1.0)	5.8 (0.8)	5.1 (1.3)
	Control of learning	5.5 (0.7)	5.5 (0.7)	5.7 (0.8)	5.1 (0.9)
	Self-efficacy	5.5 (0.8)	5.2 (0.8)	5.8 (0.8)	5.2 (0.9)

*Groups differed on the sequence of module format exposure, but all participants in each group were assigned to complete two modules in each format.

### Impact of intervention on motivation, knowledge, and time

The change in task value motivation from pre-module to post-module, as measured by the short motivation inventory, was significantly higher for the motivationally-enhanced format (standard format mean change −0.01, enhanced format mean change +0.09, difference = 0.10, CI 0.001 to 0.19; p = 0.048). The mean posttest knowledge score did not differ significantly between the standard and motivationally enhanced modules (standard mean 72.8, enhanced mean 73.0, difference = 0.2, CI −1.9 to 2.1; p = 0.90). The self-reported time to complete each module and perceived mental effort were also similar between formats; see Table 3.

**Table 3:** **Summary of between-format differences**

	Standard format	Enhanced format	Difference (95% CI)	p
	Mean (SE)	Mean (SE)		
Task value motivation (change from pretest to posttest)	−0.01 (0.05)	+0.09 (0.05)	0.10 (0.001 to 0.02)	0.048
Posttest knowledge score (% correct)	72.8 (1.1)	73 (1.1)	0.2 (−1.9 to 2.1)	0.90
Time to complete (minutes)	58.1 (2.7)	59.7 (2.7)	1.6 (−2.0 to 5.1)	0.38
Mental effort (1 = low, 7 = high)	4.60 (0.07)	4.63 (0.07)	0.03 (−0.10 to 0.17)	0.64

SE = standard error of the mean.

### Association between motivation and knowledge scores

Across all modules, a one-point increase in the pretest short motivation inventory (just prior to each module) was associated with a 2.1-point increase in posttest knowledge (b = 2.1, p = 0.003). There were no statistically significant relationships between the baseline MSLQ and posttest motivation or knowledge scores.

### Course evaluation and learner preferences

On the post-course evaluation, residents had no significant preference for module type (mean 3.4, CI 3.1 to 3.7, p = 0.63 compared with the scale median, N = 63). They also felt the two modules were similarly motivating (mean 4.1, CI 3.7 to 4.4, p = 0.72), efficient (mean 3.8, CI 3.5 to 4.2, p = 0.33), and effective (mean 4.0, CI 3.7-4.3, p = 1).

## Discussion

We hypothesized that an intervention designed to enhance motivation by having the residents imagine themselves in a supervisory role would improve both motivation and knowledge. In this randomized trial we confirmed a statistically significant improvement in task value motivation scores for the enhanced modules, but the impact on knowledge scores was small and not statistically significant. We also found an association between module-specific motivation and post-module knowledge test scores. Post-course evaluation results are limited by low response rates (which could bias results), but suggest the two modules were similar with respect to perceived effectiveness and efficiency.

The learners with higher motivation in this study had better test scores, which is consistent with previous research demonstrating associations between task value, course enjoyment, and exam results [13]. One could argue that resident trainees, by nature of their chosen career path, have a strong motivation to learn at baseline. However, it has been suggested that negative achievement emotions can still impact performance in this type of learner [12]. As such, baseline motivation may be a target to improve learning. It is possible that if a motivational module to enhance expectancy and task value preceded a learning module, the learner could engage in positive motivational emotions before a learning activity or knowledge test. Certain motivational teaching behaviors may enhance motivation [7], but a formal motivation curriculum and its effect on learning has not been explored. An intervention to increase baseline motivation could ultimately be effective for knowledge acquisition.

It is notable that topic-specific motivation (measured by the pre-module motivation inventory) was associated with improved knowledge posttest scores. By contrast, and contrary to our earlier findings [22], overall motivation measured by the MSLQ was not associated with knowledge scores. This suggests that motivation to learn may be topic-specific; for example, a learner might be more motivated to learn about hypertension than COPD. Future efforts to adapt instructional design may choose to target topic-specific motivation rather than general motivation; for example, having a learner commit to individual learning goals before a module may enhance self-efficacy and improve motivation to learn a specific topic.

Motivation was enhanced in this study by having learners imagine themselves in a supervisory role. This finding supports the idea that supervisory or teaching activities can stimulate motivation [15,17]. Since lower performing students have lower task value and self-efficacy beliefs [12], supervisory or teaching activities may be particularly important when trying to boost motivation in struggling students.

Although theories of learning and limited evidence suggest that teaching and supervisory roles should enhance learning [15-20], we failed to find evidence to support that hypothesis. Most likely, asking residents to imagine themselves in a supervisory role lacked the authenticity and urgency required to stimulate learning. Future studies could potentially enhance task value by having the virtual supervisor explain to the medical student why learning a specific module topic might be important during residency training; this could be done within a learning module or within self-assessment questions. Alternatively, a higher stakes virtual teaching role with higher acuity patients (e.g. a senior resident supervising inpatient rounds) may improve the task value of the intervention. It is also possible that learning in this study was enhanced but that the timing or measurement of this outcome was inadequate to detect the difference.

Task value motivation was the measure for this study, however, changes in other factors (e.g. feelings of well-being, fatigue, symptoms of burn-out) were not measured. There is some conflicting evidence on whether measures of well-being are associated with medical knowledge and motivation to learn [13,24]. A future study could integrate measures of well-being into its motivational assessments.

The present findings indicate that an instructional variation can influence motivation and that motivation is associated with performance. While certainly not definite, these findings show some promise and suggest the need for further research in the area of motivationally-directed instructional design.

### Limitations

Although there was a significant improvement in motivation, posttest knowledge scores were similar between module formats. We note that well-done negative studies contribute to the field by demonstrating what does not work in a particular situation. It is unlikely that we missed an important effect due to inadequate power, since we achieved our target sample size, and since the confidence intervals surrounding the observed difference exclude our a priori meaningful difference. This study was conducted in a single academic institution, and the baseline motivational characteristics of these participants may not reflect those of internal medicine and family medicine residents nationwide. If the residents in our sample had higher baseline motivation, it could lead to a ceiling effect (i.e., smaller potential difference between residents). As noted above, the virtual nature of the supervisory role lacked the authenticity and urgency of a real-life supervisory experience.

Additional strengths of this study included minimizing allocation bias by randomization and using motivation and knowledge assessment instruments whose scores had been validated for use in this learner population.

## Conclusions

The findings that putting residents into a supervisory role may increase motivation, and that higher levels of motivation are associated with better test scores suggests that motivation may prove a viable target for future efforts to improve learning. Topic-specific motivation may be more amenable to enhancement than overall motivation. Further investigation into the relationship between motivational enhancements and knowledge outcomes is warranted.

## References

[1] Kusurkar RA, Croiset G, Mann KV, Custers E, Ten Cate O (2012). Have motivation theories guided the development and reform of medical education curricula? A review of the literature. Acad Med.

[2] de Lange P, Suwardy T, Mavondo F (2003). Integrating a virtual learning environment into an introductory accounting course: determinants of student motivation. Account Educ: Int J.

[3] Barber LK, Bagsby PG, Grawitch MJ, Buerck JP (2011). Facilitating self-regulated learning with technology: evidence for student motivation and exam improvement. Teach Psychol.

[4] Choi HJ, Johnson SD (2005). The effect of context-based video instruction on learning and motivation in online courses. Am J Dist Educ.

[5] Baldwin TT, Magjuka RJ, Loher BT (1991). The perils of participation: effects of choice of training on trainee motivation and learning. Pers Psychol.

[6] Parker LE, Lepper MR (1992). Effects of fantasy contexts on children’s learning and motivation: making learning more fun. J Pers Soc Psychol.

[7] Kusurkar RA, Croiset G, Ten Cate OTJ (2011). Twelve tips to stimulate intrinsic motivation in students through autonomy-supportive classroom teaching derived from self-determination theory. Med Teach.

[8] Eccles JS, Wigfield A (2002). Motivational beliefs, values, and goals. Annu Rev Psychol.

[9] Pintrich PR (1999). The role of motivation in promoting and sustaining self-regulated learning. Int J Educ Res.

[10] Bong M (2001). Role of self-efficacy and task-value in predicting college students’ course performance and future enrollment intentions. Contemp Educ Psychol.

[11] Lau S, Liem AD, Nie Y (2008). Task- and self-related pathways to deep learning: the mediating role of achievement goals, classroom attentiveness, and group participation. Br J Educ Psychol.

[12] Artino AR, Hemmer PA, Durning SJ (2011). Using self-regulated learning theory to understand the beliefs, emotions, and behaviors of struggling medical students. Acad Med: J Assoc Am Med Coll.

[13] Artino AR, La Rochelle JS, Durning SJ (2010). Second-year medical students’ motivational beliefs, emotions, and achievement. Med Educ.

[14] Artino AR, Holmboe ES, Durning SJ (2012). Can achievement emotions be used to better understand motivation, learning, and performance in medical education?. Med Teach.

[15] Merrill MD (2013). First principles of instruction : assessing and designing effective, efficient, and engaging instruction.

[16] Dandavino M, Snell L, Wiseman J (2007). Why medical students should learn how to teach. Med Teach.

[17] Sobral DT (2004). What kind of motivation drives medical students’ learning quests?. Med Educ.

[18] First LR, Lauerman R, Fenton T, Herzog L, Snyder JD (1992). Learning by teaching. A resident-taught oral therapy program for acute diarrhea. Clin Pediatr (Phila).

[19] Apter A, Metzger R, Glassroth J (1988). Residents’ perceptions of their role as teachers. J Med Educ.

[20] Busari JO, Prince KJ, Scherpbier AJ, Van Der Vleuten CP, Essed GG (2002). How residents perceive their teaching role in the clinical setting: a qualitative study. Med Teach.

[21] Pintrich PR, Smith DAF, Garcia T, McKeachie WJ (1993). Reliability and predictive validity of the motivated strategies for learning questionnaire (MSLQ). Educ Psychol Meas.

[22] Cook DA, Thompson WG, Thomas KG (2011). The motivated strategies for learning questionnaire: score validity among medicine residents. Med Educ.

[23] Cook DA, Thompson WG, Thomas KG, Thomas MR (2009). Lack of interaction between sensing-intuitive learning styles and problem-first versus information-first instruction: a randomized crossover trial. Adv Health Sci Educ.

[24] West CP, Shanafelt TD, Cook DA (2010). Lack of association between resident doctors’ well-being and medical knowledge. Med Educ.

